# OGFOD1: a critical mediator of chemoresistance in acute myeloid leukemia

**DOI:** 10.3389/fphar.2026.1809489

**Published:** 2026-04-20

**Authors:** Tingting Qiu, Peiyi Li, Liquan Tan, Shuang Wu, Qunli Luo

**Affiliations:** 1 Department of Hematopathology, The Affiliated Changsha Central Hospital, Hengyang Medical School, University of South China, Changsha, China; 2 Respiratory Intensive Care Unit, The Affiliated Changsha Central Hospital, Hengyang Medical School, University of South China, Changsha, China; 3 Department of Nursing, The Affiliated Changsha Central Hospital, Hengyang Medical School, University of South China, Changsha, China

**Keywords:** AML, BCAA, OGFOD1, protein synthesis, translation

## Abstract

2-oxoglutarate-and iron-dependent oxygenase domain-containing protein 1(OGFOD1) is a prolyl hydroxylase that plays a pivotal role in regulating protein synthesis accuracy and efficiency. OGFOD1 is implicated in the pathogenesis of various cancers, including lung cancer, breast cancer and colonic cancer. A recent study highlights that under chemotherapy pressure, the upregulation of OGFOD1 promotes global protein synthesis via its catalytic activity, which is a key mechanism for AML chemoresistance. Deleting of OGFOD1 specifically compromises AML translation adaptability, thereby eradicating post-chemotherapy resistant cells and extending survival *in vivo*, while sparing normal hematopoiesis. Consequently, targeting OGFOD1 with identified inhibitors presents a promising therapeutic strategy to disrupt the translational adaptability of AML cells, potentially overcoming chemoresistance and improving patient outcomes. More importantly, there is an urgent need to develop highly selective OGFOD1-targeted agents with minimal toxicity.

## Introduction

2-oxoglutarate-and iron-dependent oxygenase domain-containing protein 1(OGFOD1) is a prolyl hydroxylase, with well-conserved homologs from yeast to humans. OGFOD1 is mainly located in the cytoplasmic ribosomes and binds to the 40S subunit, sparsely distributed in mitochondria (NIH, 2018). The molecular structure of OGFOD1 comprises an N-terminal catalytic domain, a nuclear localization sequence, and a C-terminal protein-interaction domain. Its core catalytic activity is dependent on Fe^2+^, 2-oxoglutarate, and molecular oxygen, and it specifically catalyzes the hydroxylation of Pro62 within the 40S ribosomal subunit protein RPS23 (uS12) (Al-Qahtani et al., 2015).

In normal cells, OGFOD1 participates in several physiological functionsincluding protein synthesis, translational regulation, eIF2αphosphorylation, stress granules formation and cell cycle regulation. Specifically, OGFOD1 enhances the fidelity and efficiency of protein synthesis by hydroxylating of the small ribosomal protein S23 (RPS23), which optimizes the accuracy of tRNA anticodon matching with coding mRNA (Besarab et al., 2015). Furthermore, OGFOD1 senses extracellular cues such as oxidative stress and nutrient deprivation, undergoes nucleocytoplasmic shuttling, and facilitates stress granule assembly to protect the translational machinery (Brillante et al., 2024). Research has shown that the overexpression of OGFOD1 increases the abundance of phosphorylated eIFαin both unstressed and arsenite-stressed cells, as well as accelerating apoptosis during stress (Cui et al., 2025). Additionally, OGFOD1 sustains normal cell cycle rhythms by indirectly regulating the transcriptional and post-transcriptional stability of cell cycle-related genes (Fets et al., 2022). As a member of the ribosomal oxygenases (ROXs) subfamily, the enzymatic activity and subcellular localization of OGFOD1 constitute the core of its physiological functions, and also serve as the key targets for its dysregulation in tumors (Fets et al., 2022).

The expression pattern and catalytic function of OGFOD1 are closely associated with the clinical characteristics and patient survival outcomes of various diseases. Bioinformatic analyses and clinical sample detections revealed that OGFOD1 expression was significantly elevated in breast cancer tissues compared with normal breast tissues. Among 1,115 breast cancer patients, 28.3% (316 cases) exhibited high OGFOD1 expression, and these patients had a significantly poorer prognosis (Fujisaki et al., 2023). The transcript and protein expression levels of OGFOD1 in chronic lymphocytic leukemia (CLL) cells and normal B cells are both higher than those in normal peripheral blood mononuclear cells. Furthermore, 5 out of 12 CLL patients who developed graft-versus-leukemia (GvL) responses after donor lymphocyte infusion (DLI) showed serologic reactivity to OGFOD1, indicating that OGFOD1 is a key immune target-related molecule in CLL patients with GvL responses following DLI treatment (Harris et al., 2022). OGFOD1 demonstrated a substantial correlation with transcriptomic characteristics in castration-resistant prostate cancer (CRPC). Therefore, potential therapeutic targets for facilitating immunotherapy in prostate cancer might be represented by OGFOD1 (Horita et al., 2015).

Bioinformatic analysis of OGFOD1-knockout mice hearts revealed a 3.5-fold enrichment of inosine 5′-monophosphate (IMP), and a 1.7-fold enrichment of β-alanine in the cardiac tissue. These alterations reduced the myocardial infarct size by 41.4% and improved cardiac function by 34%, thereby alleviating myocardial injury, myocardial infarction and other cardiac pathological changes (Katz et al., 2014). Additionally, OGFOD1 catalyzes RPS23 hydroxylation to maintain translation fidelity and proteostasis. The dysregulation of OGFOD1 promotes abnormal proliferation and cellular senescence, thereby contributing to tumorigenesis and cancer progression (Kim et al., 2015).

**FIGURE 1:**
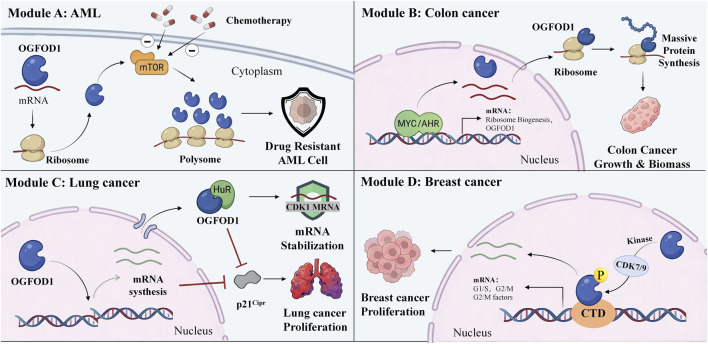
OGDFOD1: A Central Mediator of Multimodal Oncogenesis. Module, Module **(A)** Under the conditions of BCAA and 5 + 3, the translation level of OGFOD1 increased, protein synthesis increased, accompanied by elevated mTOR activity, and this further promoted the drug resistance of AML cells. Module **(B)** in colonic cells, MYC induces the expression of the transcription factor AHR, and the activated AHR can specifically initiate the transcription of target genes related to ribosome biogenesis and OGFOD1. OGFOD1 is positively regulated by AHR, participates in ribosome biogenesis and protein translation, and maintains global protein synthesis. Module **(C)** OGFOD1 mediates the transcriptional activation of CDK2 and CCNB1, thereby ensuring the normal synthesis of their mRNAs. In parallel, OGFOD1 cooperates with HuR to stabilize CDK1 mRNA at the post-transcriptional level, thus sustaining constitutive CDK1 expression, which restrains nuclear accumulation of p21Cip1 and further promotes lung cancer proliferation. Module **(D)** in breast cancer cells, OGFOD1 positively governs the mRNA levels of core transcription factors governing G1/S phase transition and G2/M phase progression to maintain orderly cell cycle progression. CDK7/9 phosphorylates serine 256 of OGFOD1. Phosphorylation-activated OGFOD1 directly binds to the CTD, modulates its phosphorylation profile and enhances its transcriptional activity, thereby driving the proliferation of breast cancer cell. BCAA: branched-chain amino acid; 5 + 3: 5 days of using cytarabine, followed by 3 days of using doxorubicin; OGFOD1: 2-oxoglutarate-and iron-dependent oxygenase 1; mTOR: mammalian target of rapamycin; AML: Acute Myelocytic Leukemia; MYC: myelocytomatosis oncogene; AHR: aryl hydrocarbon receptor; CDK1: cyclin-dependent kinase 1; CDK2: cyclin-dependent kinase 2; CCNB1: cyclin B1; p21Cip1: cyclin-dependent kinase inhibitor 1A; CDK7: cyclin-dependent kinase 7; CDK9: cyclin-dependent kinase 9; CTD: carboxyl-terminal domain.

Aberrant expression of OGFOD1 in the occurrence and development of various cancers (Figure 1). For example, OGFOD1 maintains the expression homeostasis of cell cycle-associated genes in lung cancer cells via a dual regulatory mechanism. Specifically, OGFOD1 directly mediates the transcriptional activation of cyclin-dependent kinase (CDK)2 and cyclin B1 (CCNB1) to ensure the normal synthesis of their mRNAs. In parallel, OGFOD1 cooperates with the Human antigen R (HuR) to stabilize CDK1 mRNA at the post-transcriptional level, thus sustaining the constitutive expression of CDK1. OGFOD1 knockdown blocks transcription to reduce CDK2 and CCNB1 mRNA levels, and triggers time-dependent CDK1 mRNA degradation. Meanwhile, OGFOD1 knockdown promotes nuclear accumulation of p21Cip1 to further suppress cyclin-dependent kinase activity (Fets et al., 2022). Summarily, OGFOD1 is essential for driving lung cancer cell proliferation.

In breast cancer cells, OGFOD1 positively governs the mRNA levels of core transcription factors governing G1/S phase transition and G2/M phase progression to maintain orderly cell cycle progression. Depletion of OGFOD1 drastically downregulates the mRNA expression of cell cycle-related transcription factors. These downregulation directly inhibiting G1/S transition and arresting G2/M progression and DNA compaction via accumulation of H4K20me3 at mitosis, thereby inducing repressing the proliferative capacity of breast cancer cells (Fujisaki et al., 2023). As a pivotal oncogenic driver in breast cancer, OGFOD1 facilitates malignant progression through the cell cycle-dependent kinase 7/9(CDK7/9) triggered phosphorylation axis. Specifically, CDK7/9 phosphorylates serine 256 of OGFOD1, this post-translational modification is essential for its pro-tumorigenic function, given that non-phosphorylatable OGFOD1 mutants abolish oncogenic activity. Phosphorylation-activated OGFOD1 directly binds to the C-terminal domain of RNAPII, modulates its phosphorylation profile and enhances its transcriptional activity, thereby driving the proliferation of breast cancer cells (Lafita-Navarro et al., 2018). Notably, ablation of OGFOD1 represses RNAPII-mediated transcription, ultimately restraining breast cancer cell proliferation and *in vivo* tumorigenesis, which further corroborates its feasibility as a promising therapeutic target for breast cancer.

In colonic cells, MYC induces the expression of the transcription factor aryl hydrocarbon receptor (AHR), and the activated AHR can specifically initiate the transcription of target genes related to ribosome biogenesis and OGFOD1. OGFOD1 is positively regulated by AHR, participates in ribosome biogenesis and protein translation, and maintains global protein synthesis. The SUnSET assay confirmed that silencing AHR or OGFOD1 can significantly reduce protein synthesis levels, suggesting that targeting OGFOD1-related pathways could serve as a novel strategy to limit biomass production in MYC-driven tumors (Lee et al., 2021). Recently, Mayerhofer et al. (Liesveld et al., 2022) demonstrated that OGFOD1 regulates Acute myeloid leukemia (AML) chemotherapy resistance by regulating protein synthesis.

In order to clarify the specific mechanism of AML chemotherapy resistance, Mayerhofer et al. (Liesveld et al., 2022) conducted induction chemotherapy (5 days cytarabine and 3 days doxorubicin, the 5 + 3 regimen) on chemosensitive and chemoresistant hAML patient-derived xenograft models (PDXs) in NOD scid gamma (NSG) mice, and observed a significant increase in branched-chain amino acid (BCAA) valine and leucine in chemoresistant PDX, which was related to the increased expression of BCAA transport proteins and the decreased catabolism of BCAAs. An increase in BCAA levels is associated with the drug resistance of AML cells (Marina et al., 2010). Furthermore, they collected bone marrow samples from 14 AML patients and found that the BCAA levels in leukemia stem cells (LSCs) were elevated, which is related to the resistance of LSCs to chemotherapy. That means the BCAA metabolism might be the mechanism of drug resistance in AML.

Further, the efficacy of BCAA restriction was verified in the MLL-AF9 and HoxA9-Meis1 mouse AML models. Unexpectedly, residual AML cells under combined BCAA restriction and chemotherapy exhibited markedly increased global protein synthesis *in vivo*, accompanied by elevated mTOR activity. Furthermore, the BCAA restriction when combined with the 5 + 3 chemotherapy regimen, substantially reduces residual leukemic cells in the bone marrow, achieving near-complete clearance in the HoxA9-Meis1 model. This combined approach also significantly extended the survival of leukemic mice. Notably, BCAA restriction alone demonstrates efficacy comparable to the 5 + 3 chemotherapy. These results suggest that BCAA restriction enhances the efficacy of chemotherapy.

Subsequently, they conducted Ribo-seq and Ribo-Lite analyses to assess protein synthesis in AML cells post-chemotherapy. The measurements indicated a notable increase in global translation efficiency, accompanied by elevated synthesis of ribosomal proteins. However, BCAA deprivation and chemotherapy trigger ribosome pausing at stop codons, whereas OGFOD1 translation is strongly upregulated to restore translation efficiency. This elevation overall translation efficiency, enabling AML cells to continue protein synthesis under dual stress from both nutritional deprivation and drug treatment.

To validate the function of OGFOD1, OGFOD1 KO in hAML cell lines was assessed by polysome profiling and protein synthesis assays, showing higher polysome peaks (ribosome pausing) and reduced OPP incorporation (diminished translation), indicating that OGFOD1 knockout increased ribosome pausing and reduced protein synthesis. Catalytically inactive OGFOD1 mutants (D157A, H155A) were generated and reintroduced into OGFOD1 KO cells alongside WT OGFOD1, with mutants failed to rescue AML proliferation or protein synthesis. Furthermore, the overexpression of mutant OGFOD1 reversed the increased proliferation and OPP incorporation induced by the WT OGFOD1, demonstrating that OGFOD1 catalytic activity is essential for supporting AML growth and protein synthesis.

For exploring the function of OGFOD1 in hematopoietic processes, they transplanted transduced hematopoietic stem cells (HSCs) into OGFOD1 KO mice, and observed that OGFOD1 depletion exerted a minimal effect on hematopoiesis. Then, they performed an *in vivo* rescue experiment with OGFOD1 suppression under BCAA-free plus 5 + 3 conditions. OGFOD1 deletion partially reversed the increased protein synthesis induced by BCAA restriction and chemotherapy, and altered the translational regulation of mTOR pathway components and chromatin remodeling-related genes. Therefore, targeting OGFOD1 represents a potential therapeutic strategy for chemoresistant AML.

This study has multiple critical limitations in investigating the role of OGFOD1 in regulating AML. In mechanism exploration, the research fails to directly verify the core catalytic function of OGFOD1, the hydroxylation modification of RPS23, and to clarify the upstream and downstream regulatory relationship and feedback loop between OGFOD1 and the mTOR pathway. In clinical validation, the study enrolls a small sample of human AML patients, which makes it impossible to verify the clinical correlation between relevant indicators and chemotherapy resistance or relapse. OGFOD1-related indicators in the bone marrow of drug-resistant patients are not detected, leading to a lack of direct evidence for the correlation between OGFOD1 and patient survival. In terms of therapy and safety assessment, the combined effects of OGFOD1 inhibition with the 5 + 3 chemotherapy regimen and BCAA restriction are only verified, but not with the first-line clinical VenAza (Venetoclax + Azacitidine) regimen. In addition, long-term animal safety evaluation is not conducted, and the impact of OGFOD1 inhibition on vital organs such as the liver and kidney remains unclear.

However, OGFOD1 has been preliminarily confirmed as a target for regulating chemotherapy resistance in AML, research of related inhibitors have also made certain progress, including FG4592, nitisinone, N-oxalylglycine (NOG) and miR-1224-5p. FG4592, a prolyl hydroxylase domain protein (PHDs) inhibitor. FG4592 inhibits OGFOD1 activity by directly binding to this 2-oxoglutarate-dependent non-heme iron dioxygenase in a structure-dependent manner. This specific targeted binding further blocks the OGFOD1-mediated prolyl hydroxylation of ribosomal protein RPS23 at the Pro-62 residue, thereby activating the unfolded protein response and autophagy (Mayerhofer et al., 2025). In terms of limitations, FG4592 is a non-specific inhibitor, lacking specificity for OGFOD1. This non-specificity may lead to off-target effects, as FG4592 can activate the HIF signaling pathway while inhibiting OGFOD1, thereby interfering with normal oxygen-sensing and iron metabolism processes in normal cells (Singleton et al., 2014). Moreover, FG4592 shows poor tissue targeting, which may result in insufficient drug concentration at tumor sites and reduced inhibitory effects on OGFOD1, while increasing the risk of systemic toxicity.

Nitisinone, a clinical medication primarily used to treat tyrosinemia type I. Nitisinone is regarded as an inhibitor of OGFOD1 and has been shown to affect protein synthesis (Thinnes et al., 2019). Notably, the primary canonical target of nitisinone is 4-hydroxyphenylpyruvate dioxygenase (HPPD) rather than OGFOD1. Furthemore, nitisinone lacks tailored affinity for the catalytic active site of OGFOD1. Regarding potential toxicities, the most pronounced adverse effect is severe hypertyrosinemia, which complicates the clinical use of nitisinone for OGFOD1 inhibition (Wehner et al., 2010).

NOG is a broad-spectrum 2-OG oxidase inhibitor that blocks the catalytic activity of OGFOD1 by occupying its active site (Al-Qahtani et al., 2015). NOG blocks the entire family of 2OG-dependent non-heme iron dioxygenases, resulting in widespread off-target effects (Wu et al., 2023). Additionally, NOG has poor cell permeability and low metabolic stability in biological systems (Xie et al., 2024), requiring high working concentrations to achieve partial OGFOD1 inhibition.

miR-1224-5p is a member of the microRNAs (miRNAs) family that can suppress the expression of OGFOD1, thus suppress cell proliferation and induces apoptosis (Yin et al., 2020). As a post-transcriptional inhibitor targeting OGFOD1, miR-1224-5p faces critical delivery-related limitations. Naked microRNA is rapidly degraded by nucleases in biological fluids, resulting in low effective concentration at target sites (Zhuang et al., 2015). Therefore, several inhibitors of OGFOD1 have undergone basic functional validation, yet the existing ones are commonly plagued by inadequate target specificity, low delivery efficiency and prominent safety risks. Meanwhile, such inhibitors lack clinical trial data, verification of combination with first-line therapeutic regimens and long-term safety evaluation, which severely hinders their translational progress from the laboratory to clinical practice.

In summary, OGFOD1 regulates protein synthesis, enabling AML cells to survive under chemotherapy and nutrient deficiency. Targeting OGFOD1 specifically compromises AML translation adaptability, thereby eradicating post-chemotherapy resistant cells and extending survival *in vivo*, while sparing normal hematopoiesis. Hence, drugs targeting for OGFOD1 may open a new pathway of therapeutic intervention for chemoresistant AML. Notably, as there are no highly specific and safe inhibitors available for the targeted therapy of OGFOD1-related malignancies. There is an urgent need to develop highly selective OGFOD1-targeted agents with minimal toxicity to advance mechanistic studies.

## Data Availability

The original contributions presented in the study are included in the article/supplementary material, further inquiries can be directed to the corresponding author.
